# A Thought-Operated Digital Random-Access Memory

**DOI:** 10.1155/2019/9684140

**Published:** 2019-06-09

**Authors:** Lee Ben-Ami, Ido Bachelet

**Affiliations:** ^1^Augmanity, Rehovot, Israel; ^2^Faculty of Life Sciences, BIU, Ramat Gan, Israel; ^3^Gonda Brain Centar, BIU, Ramat Gan, Israel

## Abstract

The capacity and reliability of biological memory could be exceeded by a constantly growing flux of information to remember and operate by. Yet, our memory is fragile and could be easily impaired, and the prevalence of memory disorders is increasing in correlation with the population's mean age. As expected, auxiliary memory devices (such as writing pads and computers) are abundant but are operated indirectly using significant effort compared with biological memory. We report a working prototype of a simplified, 4 KB random-access memory (RAM) that can be written to or read from using thought and could be embedded more seamlessly than other artificial memory aids. The system analyses EEG signals to extract attention levels, which trained subjects can use to write messages into an RFID sticker, or read from it on a display. We describe basic modes of using memory by a single subject, emulate common forms of social communication using this system, and highlight new forms of social usage and allocation of memories that are linked to specific persons. This preliminary prototype highlights the technical feasibility and the possibilities of implantable thought-operated memory devices and could be developed further to provide seamless aid to people suffering from memory disorders in the near future.

## 1. Introduction

Our ability to store and retrieve information is critical for learning, social interaction, and experience and hence for our survival [1]. However, it is also a fragile faculty and could be damaged or lost relatively easily. Memory disorders and dementia, which are hallmarks of medical conditions ranging from mild cognitive impairment (MCI) to Alzheimer's disease (AD), are a significant problem which is growing steadily [2–4], for which treatment is extremely limited and inadequate [5]. Our ability to create short-term or long-term memories could be severely damaged by head trauma, infarcts, diseases, and even the side effects of certain drugs [6].

On the other hand, biological memory could be assisted by simple means such as external documentation, e.g., by writing and audio recording. However, two challenges could be anticipated. First, the amount of information that we come across and are required to remember properly is constantly increasing, for example, the number of individuals that we need to maintain direct contact with. Excessive flow of information could hinder the task of indirectly documenting this information. Second, auxiliary memory devices such as a notebook or mobile phone could be easily lost, stolen, or damaged and are thus of limited reliability. Information can be stored on a database which is accessible everywhere, such as a cloud, but this access requires network connectivity, which is still largely limited and discontinuous.

We could therefore envision an auxiliary memory device which is direct, in order to allow seamless documentation and retrieval of information, and has the ability to be embedded or implanted, as to reduce the chances for random loss of the memory stored on it. Such auxiliary memory could function in parallel to our native capacity to remember as a backup or failsafe system that comes into action when needed.

Comparison to other studies of artificial memory devices shows prototypes like the implanted silicon chip in the work of Berger et al. [7] that was implanted in rats and monkeys and can process information similar to actual neurons. This chip does not store data but can serve as a prosthesis for a damaged part in the hippocampus. Another surgical implant used for patients with hearing disabilities is the Cochlear implant which provides a sense of sounds with an electric device stimulating the auditory nerve [8]. This device does not store or share data but is used to convert sound waves into an electrical stimulus of the nerve. Another study was done by Sum-Gyun Yi et al. which fabricated MoS2-based flash memory devices by stacking MoS2 and hexagonal boron nitride (hBN) layers on an hBN/Au substrate and demonstrated that these devices can emulate various biological synaptic functions, including potentiation and depression processes, spike-rate-dependent plasticity, and spike-timing dependent plasticity [9]. This fabricated memory mimics the work of a synapse in a specific brain. These studies represent a variety of implant studies in the field of implants regarding memory or neural abilities. All are invasive and do not store or share data.

Another era of study deals with how psychology works or cultures created collective memory in history. These studies argue on humans' abilities to work together and using language as a mean of collaboration [10, 11]. These studies are theoretical and do not implement but show how we, as humans, collaborate and create more by using this ability.

The creation of the Internet, the cloud of data, and the Internet of things (IOT) enhance our ability to communicate wildly and store and retrieve data massively while wearing communicating devices that can monitor, store, and send data between devices and through the Internet [12, 13]. The need to help memory disabilities and IOT open the ability to create new methods of storing memory and sharing it with others thus helping and enhancing human capabilities.

In this study, we aim to do so with a new and preliminary artificial memory prototype.

**Figure 1:**
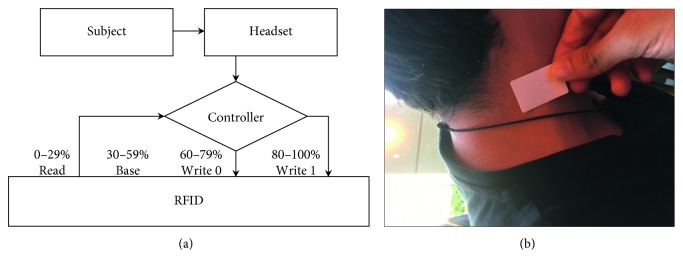
Description of the system used in this study. (a) Schematic representation. An EEG headset was used to directly acquire EEG data from the subjects. The EEG was processed and classified on a controller connected to the RFID memory circuit. Based on the attention levels measured from the subject, the controller performed a specific function on the memory: read memory content (0%–29%), no action (30–59%), write 0 (60–79%), or write 1 (80–100%). (b) The RFID memory chip as a sticker on one of the subjects of this study.

Our purpose in this study was to outline and demonstrate a working prototype of such memory device. To this end, we used simple components which were wired as depicted in Figure 1(a). A commercially available headset was used to acquire EEG signals from human subjects, and a custom-written algorithm was used to extract the level of attention exhibited by each subject as previously described [14]. A controller recorded and analyzed the data in real time, and communicated with a “memory,” based on a simple RFID tag, which was stuck on the subject's neck (Figure 1(b)). Based on their level of attention, the controller carried out one of four functions: none, write 0, write 1, or read. In the terms of this demonstration, the controller is a computer capable of communicating with the RFID tag, writing bits to it, and displaying its content, e.g., as simple text. The system here has been implemented using either commercially available Arduino parts, or a standard laptop computer.

There are several types of RFID tags [15], namely, passive, active, and semi active. In our experiment, we used a passive sticker that can be activated and communicated only by a near electric field. Moreover, there are different tags and protocols of near field communication (NFC). Here, we used a MIFARE Classic ISO/IEC 14443 Type A standard which enables 4 KB memory divided to 16 sectors. Every sector holds 4 blocks of 32 hexadecimal memory digits. The first block of every sector holds a 6-digit security key (16^6^ options) that prevents access to the data and prevents reading or writing if it is incorrect, providing a layer of security to the communication between the controller and the memory [16].

Moreover, there are different types of RFID frequencies, the lower frequency 125/134.2 kHz is useful up to 30 cm and can create distance security; however, it is less optimal for longer distances. Frequencies of 868/959 MHz (UHF) or 2400 MHz can give longer distance abilities (3 to 100 m) [17]. In our experiment, we used a 13.56 MHz RFID circuit, which supports communication at up to 1 m.

## 2. Methods

### 2.1. Subjects

For this study, 9 subjects (5 women, 4 men, ages 18–43, average age 31.7 ± 10.9 years, *σ*^2^ 118.27) were recruited. We chose controlling attention levels since it is a parameter that is well researched and tested in EEG data and already used in other works. There are some hardware and applications that already use it in different ways like computer games or for research [14, 18, 19]. The reason for the four ranges is to create different letters and mode in a language of two digits (0/1) and to differ between read, write, and no request at all as explained in Figure 1. First, each subject underwent a short (average ∼15 min) phase of training of the system until they were able to achieve specific attention of one of four levels. These levels were defined based on a scale of 0–100% attention, and each was used to code a specific function: 0–29% read from memory, 30–59% baseline for “no action” and to differ between reading and writing, 60–79% write “0” to memory, and 80–100% write “1” to memory. Each subject was allowed to achieve her/his own speed in switching between attention levels, with an average of 3.5 ± 1.2 s spent at each level at the end of the training phase. Attention levels defining the ends of the scale were achieved by experiencing passive activity versus a difficult mathematical problem as previously described [14, 18, 19]. In the testing phase, the subjects were requested to read or write 0/1 by achieving the desired level of attention described above. The study design was reviewed and approved by the Institutional Review Board at Bar-Ilan University. All methods were performed in accordance with the relevant guidelines and regulations. Informed consent was obtained from all subjects prior to participating in this study.

### 2.2. Hardware and Software

EEG data were acquired using a Neurosky *Mindwave mobile plus* kit headset that provides raw-sampled wave values (128 Hz or 512 Hz, depending on hardware), signal quality metrics, eSense attention meter values (0 to 100), and EEG band power values for delta, theta, alpha, beta, and gamma.

EEG signals were obtained from neurosky mobile algorithm analysis. The Attention meter algorithm (eSens) indicates the intensity of mental “focus” or “attention.” The value ranges from 0 to 100. The attention level increases when a user focuses on a single thought or an external object and decreases when distracted. Users can observe their ability to concentrate using the algorithm. In educational settings, attention to lesson plans can be tracked to measure their effectiveness in engaging students. In gaming, attention has been used to create “push” control over virtual objects.

eSense Attention meter indicates the intensity of a user's level of mental “focus” or “attention,” such as that which occurs during intense concentration and directed (but stable) mental activity. Its value ranges from 0 to 100. Distractions, wandering thoughts, lack of focus, or anxiety may lower the Attention meter level. For each different type of eSense (i.e., Attention and Meditation), the meter value is reported on a relative eSense scale of 1 to 100. On this scale, a value between 40 and 60 at any given moment in time is considered “neutral” and is similar in notion to “baselines” that are established in conventional brainwave measurement techniques (though the method for determining a ThinkGear baseline is proprietary and may differ from other methods). A value from 60 to 80 is considered “slightly elevated” and may be interpreted as levels tending to be higher than normal (levels of Attention or Meditation that may be higher than normal for a given person). Values from 80 to 100 are considered “elevated,” meaning they are strongly indicative of heightened levels of that eSense. Similarly, on the other end of the scale, a value between 20 and 40 indicates “reduced” levels of the eSense, while a value between 1 and 20 indicates “strongly lowered” levels of the eSense. These levels may indicate states of distraction, agitation, or abnormality, according to the opposite of each eSense [20].

The signals were broadcast via Bluetooth to a controller for processing and classification. We used an *Arduino Uno* device connected to *BlueSMiRF silver* Bluetooth antenna, which translated the signals from the mindwave mobile headset device using a custom-written code. To process and classify the signals, an additional code was written using Arduino language (based on C/C++). The base program handles the Attention signals and determines the levels to classify. An NFC (near field Communication) Reading/Writing antenna shield (13.56 MHz band) was connected to the controller. A *Mifare classic* RFID tag with a 4 KB memory storage was used to store the data written or to broadcast the data when reading. Arduino and NFC antenna shields were connected to a DELL I5–4200U (2.3 GHz/4 GB RAM) laptop with windows 7 operating system which was used as display monitor.

## 3. Results

**Figure 2:**
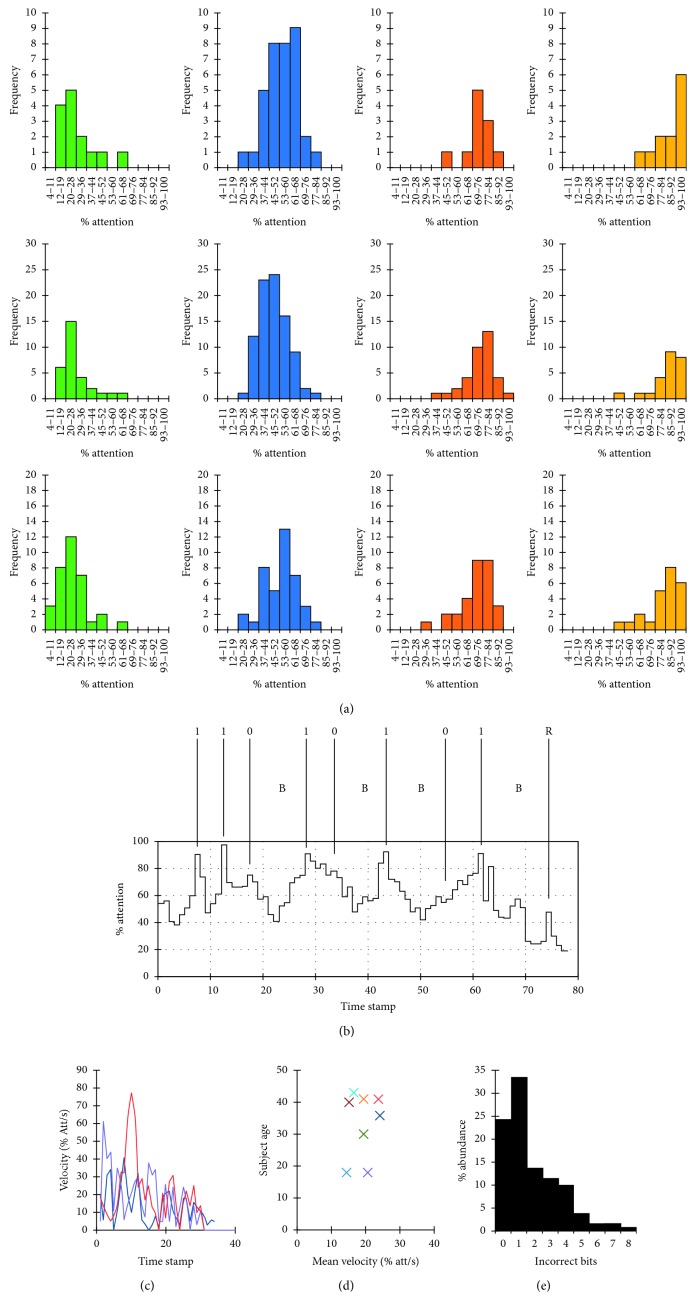
Performance of the thought-operated memory device. (a) Performance histograms of 3 representative subjects, summarizing their ability to achieve and maintain a specific attention level during 10 writing and reading tasks (green = read, blue = no action, orange = write 0, yellow = write 1); vertical coordinate, Frequency, indicates the number of times a subject reached a curtain attention level; horizontal coordinate, Attention, indicates the level of attention reached divided to sections (4–11, 12–19, 20–28, 29–36, 37–44, 45–52, 53–60, 61–68, 69–76, 77–84, 85–92, 93–100). (b) A representative memory task. Here, the subject was asked to write onto her memory the string “11010101” and then read it. This specific task was carried out without errors (25% of all tasks were error-free). (c) Velocity analysis from 3 representative short tasks, showing the transition between attention levels slowing down with time. (d) A plot of mean velocity vs. subject age, showing no correlation between these parameters. (e) Analysis of the abundance of errors in writing tasks, showing that ∼25% of the tasks were error-free, and ∼33% of the tasks had 1 incorrect bit.

Most subjects were capable of achieving desired levels of attention to be able to perform reading and writing 0/1 tasks and in a reproducible manner (Figure 2(a)). Subjects typically returned to baseline after 1 or 2 writing actions (either write 0 or write 1) and were able to maintain a maximum of 3 writing actions without returning to baseline (Figure 2(b)). Analysis of the transitions between attention levels revealed that all subjects were capable of switching rapidly between levels, achieving a velocity of up to ∼80% per second, but these transitions became slower with time (Figure 2(c)), eventually reaching a maximum velocity of 5% per second after 60 seconds of writing onto the memory. Interestingly, the ability to maintain transition efficiency did not correlate with subject age, as hypothesized at an early stage of this study, bolstering the role of training in subject performance (Figure 2(d)). Only 25% of the messages were written without incorrect bits, with most messages having 1 incorrect bit (Figure 2(e)). No bias to a specific error bit (0 or 1) was found despite the unequal allocation of attention levels to the different bits.

We used the system to investigate the possibility for social communication between individuals, mediated by writing to and reading from neighboring memories. Social communication presents a prevalent framework of communication (e.g., social networks accessed via mobile devices), which we aimed to emulate using our system. Our basic purpose was to show that the system not only supports common modes of social networking, but also allows new concepts for using memory.

**Figure 3:**
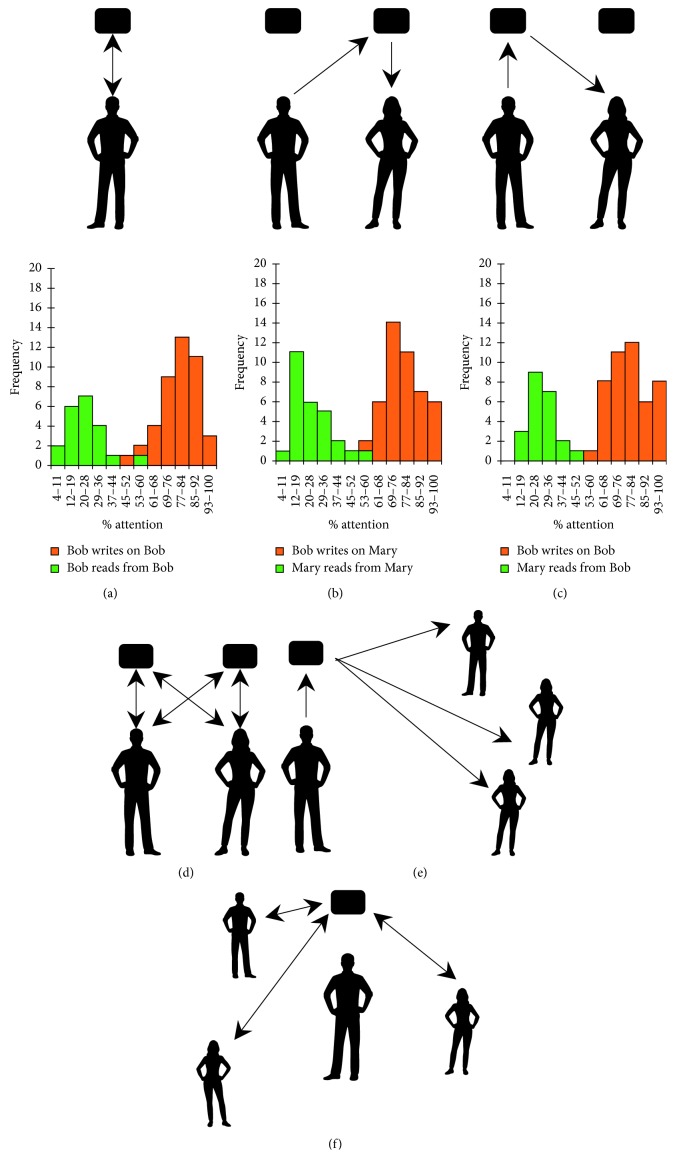
Social communication emulated with the memory device. (a–c) Three operation modes, top panels show schematic representation of the network, with histogram overlays below summarizing the reading and writing (both 0 and 1) performance achieved by the subjects during 10 tasks. Black rectangles represent memory devices. Arrow directions represent writing (arrow leading from person to device) or reading (arrow leading from device to person). (a) The basic mode of operation where same subject writes on his/her own memory and reads from it. (b) Two subjects, nicknamed Bob and Mary, emulating social communication. Bob writes onto Mary's device, and Mary reads from her device. (c) Bob writes onto his own device, and Mary reads this content from her device. (d–f) Potential modes of social usage of the memory device described here. (d) Complete sharing of memories between two subjects; (e) sharing memories from a person's memory to a public; and (f) a public authorized to use another person's memory by means of outsourcing.

In the first series of tests described above, the basic mode of operation of this device was studied: subject A writes to A (same subject) → A reads from A (Figure 3(a)). Subsequently, two subjects (generically termed Bob and Mary) used the system to write a message from Bob to Mary, by having Bob write to Mary's memory and Mary reading from her Memory (Figure 3(b)), and to emulate “mind reading,” by having Bob write to his own memory and Mary reading from Bob's memory (Figure 3(c)). These tests were handled and discussed to emphasize the potential of this work not only to store and retrieve self-memory data but in order to share memories between subjects as well. Bob reading and writing his own memory is a self-memory method. Mary reading from Bob's memory enables memory sharing from Bob's memory to Mary's.

Other modes that the system can support, although not investigated here, are sharing of memories between subjects (Figure 3(d)) or from a single person to a public (Figure 3(e)), two modes that are enabled in typical social networks today; however the system also supports the outsourcing of another person's memory (Figure 3(e)), which is not a standard social networking mode. Further designs are now being tested in our laboratory that implement different compartments, accessible by authorized individuals other than the one to whom the memory is linked, which support private allocation of information for memory outsourcing.

Although the specifications were defined arbitrarily in this system (e.g., attention levels, free pace and duration between actions), similar measurements could theoretically be made in other configurations. However, several principles were implemented in this particular design. First, the attention levels were nearly evenly distributed across the complete scale. Second, the writing actions were clustered together to enable rapid transition between them. Third, writing and reading actions were separated by the baseline range. Our findings show that the first and third principles were important in achieving reproducibility and a flowing writing uninterrupted by reading, but the second principle was less successful in ensuring that writing was not interrupted by baseline phases.

## 4. Discussion

The prototype described here is extremely preliminary in the sense that it is motivated by seamless embedding of memory without being seamless in itself. However, this is a technical barrier that is being tackled, or has been tackled successfully in some cases. RFID circuits such as the one used here are completely implantable [15, 21–23], and their interference with existing devices such as pacemakers has been studied [22]. The portability of other components of the system is being improved towards complete implants, or at least wearable or in patch form. EEG measurements themselves could be made using sensor pads or implantable sensors [24–27], eliminating the need for a carried EEG headset. Display of the content that is retrieved from the memory could be done by means of contact lens [28, 29], or, less directly, on glasses such as Google glass. Eventually, a system similar to the one described here could be entirely implantable. Moreover, the capacity of 4 KB implemented here could certainly be increased in future designs.

The specific method of writing and reading from the device could be improved. Attention is a parameter that can be readily extracted from raw EEG signals [14, 18, 19], and our observations show that trained subjects could switch between desired levels of attention sufficiently for the system to recognize the appropriate function to be carried out. However, most (∼75%) messages contained at least 1 incorrect bit. This suggests that either there is a better parameter to guide the system by or that the short training provided in this study was not sufficient. Further experiments are underway to investigate additional parameters within EEG data that could be used and to evaluate the potential precision of their utilization.

Implantable memory devices raise their own issues of privacy, possibilities for unauthorized reading, and inadvertent manipulation. Physical proximity, as required in the described prototype, is an important protective factor but limits the social applications of such devices. To enable the full scale of uses, implantable memory devices should be designed with specific layers of security addressing these special challenges, such as interference from adjacent devices and other implants and potential attacks made against the person through the implanted device.

Comparing this work with other artificial memory devices introduced earlier shows the potential of a noninvasive prototype that can be used to store and share data between 2 or more persons and to use one mind or more as a “cloud” similar to sharing thoughts and memories in social networks or the Internet today. The ability to communicate in a standard network like NFC described here may offer a connection to other devices and may correlate to other languages in future work. In contrast, converting this prototype to an invasive one as other introduced implants may give other abilities of extending human memory and brain capacity capabilities that were not found in today's implants [30].

## Data Availability

The data used to support the findings of this study are available from the corresponding author upon request.
